# The ESHRE/ESGE consensus on the classification of female genital tract congenital anomalies^†,^^‡^

**DOI:** 10.1093/humrep/det098

**Published:** 2013-06-14

**Authors:** Grigoris F. Grimbizis, Stephan Gordts, Attilio Di Spiezio Sardo, Sara Brucker, Carlo De Angelis, Marco Gergolet, Tin-Chiu Li, Vasilios Tanos, Hans Brölmann, Luca Gianaroli, Rudi Campo

**Affiliations:** 1 Congenital Uterine Malformations (CONUTA) common ESHRE/ESGE Working Group, ESGE Central Office, Diestsevest 43/0001, 3000 Leuven, Belgium; 2 First Department of Obstetrics & Gynecology, Aristotle University of Thessaloniki, Tsimiski 51 Street, Thessaloniki 54623, Greece

**Keywords:** female tract, classification system, anatomy

## Abstract

**STUDY QUESTION:**

What classification system is more suitable for the accurate, clear, simple and related to the clinical management categorization of female genital anomalies?

**SUMMARY ANSWER:**

The new ESHRE/ESGE classification system of female genital anomalies is presented.

**WHAT IS KNOWN ALREADY:**

Congenital malformations of the female genital tract are common miscellaneous deviations from normal anatomy with health and reproductive consequences. Until now, three systems have been proposed for their categorization but all of them are associated with serious limitations.

**STUDY DESIGN, SIZE AND DURATION:**

The European Society of Human Reproduction and Embryology (ESHRE) and the European Society for Gynaecological Endoscopy (ESGE) have established a common Working Group, under the name CONUTA (CONgenital UTerine Anomalies), with the goal of developing a new updated classification system. A scientific committee (SC) has been appointed to run the project, looking also for consensus within the scientists working in the field.

**PARTICIPANTS/MATERIALS, SETTING, METHODS:**

The new system is designed and developed based on (i) *scientific research* through critical review of current proposals and preparation of an initial proposal for discussion between the experts, (ii) *consensus measurement* among the experts through the use of the DELPHI procedure and (iii) *consensus development* by the SC, taking into account the results of the DELPHI procedure and the comments of the experts. Almost 90 participants took part in the process of development of the ESHRE/ESGE classification system, contributing with their structured answers and comments.

**MAIN RESULTS AND THE ROLE OF CHANCE:**

The ESHRE/ESGE classification system is based on anatomy. Anomalies are classified into the following main classes, expressing uterine anatomical deviations deriving from the same embryological origin: *U0, normal uterus; U1, dysmorphic uterus; U2, septate uterus; U3, bicorporeal uterus; U4, hemi-uterus; U5, aplastic uterus; U6, for still unclassified cases*. Main classes have been divided into sub-classes expressing anatomical varieties with clinical significance. Cervical and vaginal anomalies are classified independently into sub-classes having clinical significance.

**LIMITATIONS, REASONS FOR CAUTION:**

The ESHRE/ESGE classification of female genital anomalies seems to fulfill the expectations and the needs of the experts in the field, but its clinical value needs to be proved in everyday practice.

**WIDER IMPLICATIONS OF THE FINDINGS:**

The ESHRE/ESGE classification system of female genital anomalies could be used as a starting point for the development of guidelines for their diagnosis and treatment.

**STUDY FUNDING/COMPETING INTEREST(S):**

None.

## Introduction

Congenital malformations of the female genital tract are defined as deviations from normal anatomy resulting from embryological maldevelopment of the Müllerian or paramesonephric ducts. They represent a rather common benign condition with a prevalence of 4–7% (Grimbizis *et al*., 2001; Saravelos *et al*., 2008; Chan *et al*., 2011a). Moreover, depending on the type and the degree of anatomical distortion, they are associated with health and reproductive problems (Grimbizis *et al*., 2001, 2004; Joki-Erkkilä and Heinonen, 2003; Fedele *et al*., 2005; Strawbrigde *et al*., 2007; Mollo *et al*., 2009; Rock *et al*., 2010; Chan *et al*., 2011b; Brucker *et al*., 2011; Gergolet *et al*., 2012). Due to their prevalence and clinical importance, a reliable classification system seems to be extremely useful for their management; effective categorization enables more effective diagnosis and treatment as well as a better understanding of their pathogenesis (Grimbizis and Campo, 2010).

Until now, three systems have been proposed for the classification of female genital tract anomalies, although historically attempts for their categorization started quite earlier (Grimbizis and Campo, 2010; Acien and Acien, 2011): the American Fertility Society's (AFS) currently American Society of Reproductive Medicine system (Buttram and Gibbons, 1979; AFS, 1988), the embryological-clinical classification system of genito-urinary malformations (Acien *et al*., 2004; Acien and Acien, 2011) and the Vagina, Cervix, Uterus, Adnexae and associated Malformations system based on the tumor nodes metastases (TNM) principle in oncology (Oppelt *et al*., 2005).

Although each proposal does not have the same acceptance, with that of the AFS classification system to be higher than the others, all of them seem to be associated with serious limitations in terms of effective categorization of the anomalies, clinical usefulness, simplicity and friendliness (Grimbizis and Campo, 2010). It is noteworthy to mention that these limitations also gave place to further subdivisions for certain categories of anomalies (Rock *et al*., 1995, 2010; Joki-Erkkilä and Heinonen, 2003; Troiano and McCarthy, 2004; Strawbrigde *et al*., 2007; Gubbini *et al*., 2009; El Saman *et al*., 2012). A systematic re-evaluation of the current proposals, within a project of the European Academy for Gynecological Surgery (EAGS), has been already published underlying the need for a new and updated clinical classification system (Grimbizis and Campo, 2010).

The European Society of Human Reproduction and Embryology (ESHRE) and the European Society for Gynaecological Endoscopy (ESGE), recognizing the clinical significance of female genital anomalies, have established a common working group under the name CONUTA (CONgenital UTerine Anomalies), with the goal of developing a new updated classification system. For this purpose, a scientific committee (SC) has been appointed to run the project, looking also for consensus within the scientists working in the field through the use of DELPHI procedure (Fink *et al*., 1984; Jones and Hunter, 1995; Grimbizis and Campo, 2010).

The ESHRE/ESGE classification system of female genital anomalies is presented in this paper. It is designed having mainly clinical orientation and being based on the anatomy of the female genital tract.

## Strategy for the development of the new system

**Figure 1:**
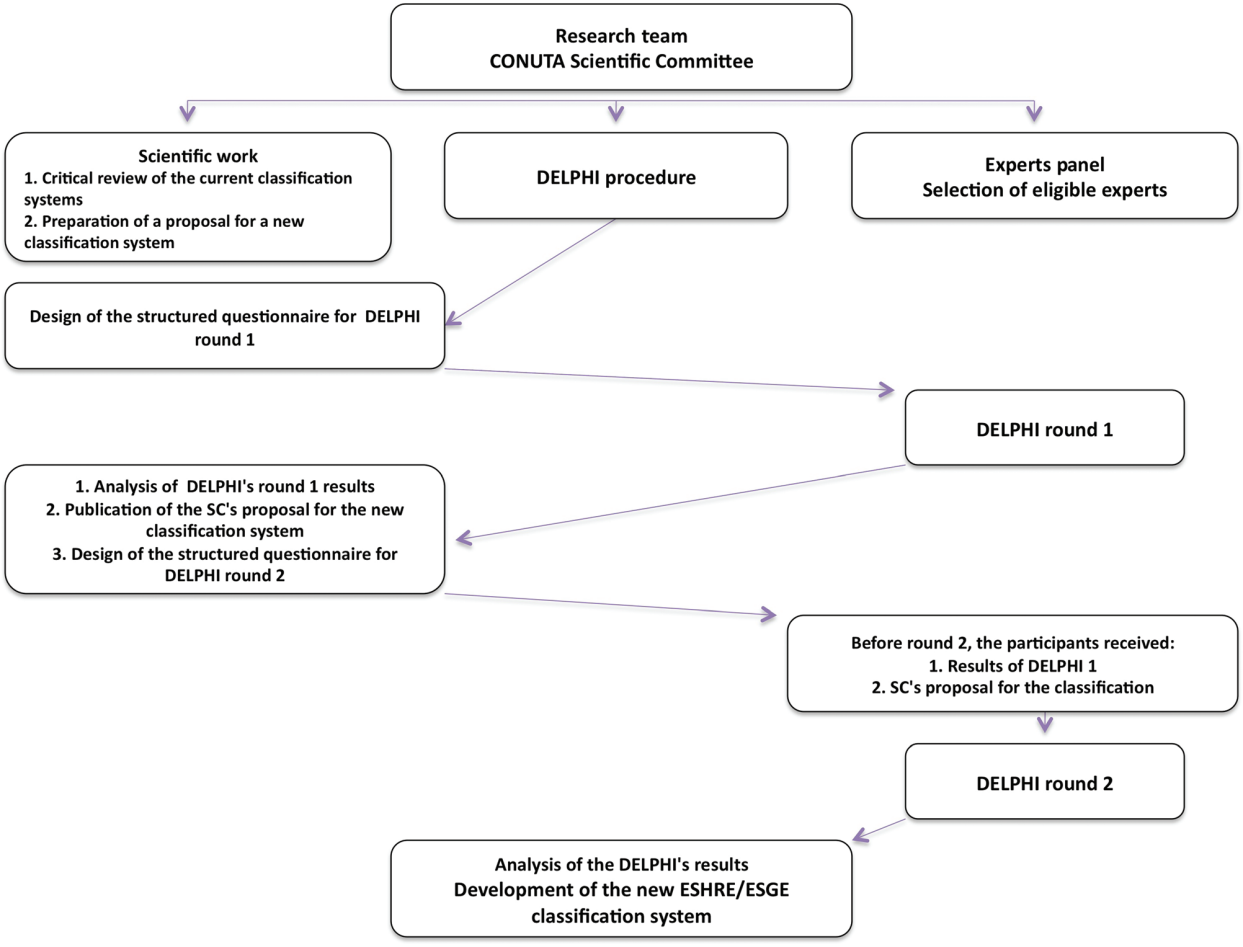
Design and running of the project; the stepwise DELPHI consensus method has been used to find the agreement between the experts in the development of the new classification system.

The development of the new ESHRE/ESGE classification system by the CONUTA ESHRE/ESGE Working Group included the following steps (Fig. 1): (i) *scientific work* for the preparation of the questionnaires, the design of the new system and the preparation of an initial proposal for discussion among the experts in the field, (ii) *consensus measurement* through the use of the DELPHI procedure to assess the extent of agreement of the experts and to have their comments for the development of the new system and (iii) *consensus development* through the incorporation of the results of the DELPHI procedure and of the comments of the experts by the SC into the final classification system.

### Scientific work for the design of the new system

Scientific work was necessary for the evidence-based development of the new classification system; it was also a prerequisite for the design of the structured questionnaires for the DELPHI procedure.

The scientific work had two distinct parts: (i) *As first part*, a systematic re-evaluation of the current proposals has been done and, based on their criticism, the characteristics of the new classification system have been clarified (Grimbizis and Campo, 2010). This work was run as a project of the EAGS and, it was later adopted by the SC of the CONUTA group. This document has been used as the scientific basis for the design of the structured questionnaire for the DELPHI procedure. (ii) The *second part* was the preparation of a proposal for the new updated clinical classification of female genital anomalies to be used during the DELPHI procedure to rank the agreement of the experts and to have their comments before deciding the final classification system. The proposal of the SC for the classification of uterine anomalies *has only been published just before the second round of the DELPHI* procedure (Grimbizis and Campo on Behalf of the CONUTA Group, 2012) in *order to have the blind answers of the experts during round one*.

### DELPHI procedure for consensus assessment

DELPHI procedure is a well-known consensus method enabling to derive quantitative estimates through qualitative approaches. It aims to rank the agreement on a scientific issue with conflicting evidence, the extent to which each participant agrees with the issue under consideration and the extent to which the responders agree with each other (Fink *et al*., 1984; Jones and Hunter, 1995; Vonk Noordegraaf *et al*., 2011).

The DEPLHI procedure for the development of the new classification system has been designed and processed into two rounds as follows:

#### Preparation phase

##### Preparation of the structured questionnaires

The SC, based on the scientific work previously described, has designed for the first round of the DELPHI procedure a structured questionnaire aiming to have the opinion of the participants on the need and the desired characteristics of a new classification system. For this reason, questions have been grouped under a limited number of headings and statements drafted for circulation to all participants (Table I). Furthermore, there was a section for comments. For the second round, the questionnaire has been changed aiming to assess the agreement of the participants with the new proposal and to have their comments on it (Tables II and III).

**Table I:** The need for and the characteristics of a new classification system: structured questionnaire for the first round of the DELPHI procedure.

**I. Is there a need for a new classification system?**				
Strongly agree	Agree	Indifferent	Disagree	Strongly disagree
42/89 (47.2%)	35/89 (39.3%)	8/89 (9%)	4/89 (4.5%)	0%
Agreement: 86.5%		9%	Disagreement: 4.5%	
***Rate of agreement: 82%***				
**II. Select the three most important characteristics that should be taken into account in the development of a new system**				
Characteristic	Ranking	Selected as one of the three		
Clear and accurate	1	**72.1%**		
Comprehensive		36.8%		
Correlated with patient's clinical presentation		39.7%		
Correlated with patient's management	2	**66.2%**		
Simple and friendly	3	**64.7%**		
Smooth movement from the old to the new one		20.6%		
**III. Which of the following statements should be taken into account in the development of a new system**				
**1. Anatomy should be the basic characteristic for patients’ grouping**				
Strongly agree	Agree	Indifferent	Disagree	Strongly disagree
51/89 (57.3%)	33/89 (37.1%)	5/89 (5.6%)	0%	0%
Agreement: 94.4%		5.6%	Disagreement: 0%	
***Rate of agreement: 94.4%***				
**2. There is a ‘key’ organ of the female genital tract that should be used in priority for the development of a new system**				
Strongly agree	Agree	Indifferent	Disagree	Strongly disagree
25/89 (28.1%)	30/89 (33.7%)	24/89 (27.0%)	9/89 (10.1%)	1/89 (1.1%)
Agreement: 61.8%		27.0%	Disagreement: 11.2%	
***In case of agreement, indicate: uterus***				
***Rate of agreement: 50.6%***				
**3. Embryological origin should be the basic characteristic for patients’ grouping**				
Strongly agree	Agree	Indifferent	Disagree	Strongly disagree
12/89 (13.5%)	13/89 (14.6%)	42/89 (47.2%)	20/89 (22.5%)	2/89 (2.2%)
Agreement: 28.1%		47.2%	Disagreement: 24.7%	
***Rate of agreement: 3.4%***				
**4. Embryological origin should be used, if feasible, as a secondary characteristic for patients’ grouping**				
Strongly agree	Agree	Indifferent	Disagree	Strongly disagree
13/89 (14.6%)	50/89 (56.2%)	18/89 (20.2%)	7/89 (7.9%)	1/89 (1.1%)
Agreement: 70.8%		20.2%	Disagreement: 9.0%	
***Rate of agreement: 61.8%***				
**IV. Comments (feel free to make additional comments)**				

The scale of answers includes five degrees to rank the agreement in each scientific issue; the extent of agreement between the participants is shown in percentages.

**Table II:** Development of the new classification system: CONUTA proposal for the classification of female genital tract malformations; it has been sent to the participants together with the results of the first round just before the questionnaire of the second round.

	Main class	Main sub-class	Co-existent sub-class
	Uterine anomaly		Cervical/vaginal anomaly
Class 0	Normal uterus		*Cervix* C0: Normal C1: Septate C2: Double ‘normal’ C3: Unilateral aplasia/dysplasia C4: Aplasia/dysplasia *Vagina* V0: Normal vagina V1: Longitudinal non-obstructing vaginal septum V2: Longitudinal obstructing vaginal septum V3: Transverse vaginal septum/imperforate hymen V4: Vaginal aplasia
Class I	Dysmorphic uterus	a. T-shaped b. Infantilis	
Class II	Septate uterus	a. Partial b. Complete	
Class III	Dysfused uterus (including dysfused ‘septate’)	a. Partial b. Complete	
Class IV	Unilaterally formed uterus	a. Rudimentary horn with cavity (communicating or not) b. Rudimentary horn without cavity/aplasia (no horn)	
Class V	Aplastic/dysplastic	a. Rudimentary horn with cavity (bi- or unilateral) b. Rudimentary horn without cavity (bi- or unilateral)/aplasia	
Class VI	Unclassified malformations

**Table III:** Development of the new classification system: structured questionnaire for the second round of the DELPHI procedure; the participants have been asked to adapt their responses taken into account the answers of the first round and the new proposal.

**I. This new classification system fulfill my needs and expectations**				
Strongly agree	Agree	Indifferent	Disagree	Strongly disagree
22/71 (31.0%)	40/71 (56.3%)	7/71 (9.9%)	1/71 (1.4%)	1/71 (1.4%)
Agreement: 87.3%		9.9%	Disagreement: 2.8%	
***Rate of agreement: 84.5%***				
**II. Please point out how far the following characteristics are addressed by the new classification**				
**1. It is clear and accurate (in definitions)**				
Strongly agree	Agree	Indifferent	Disagree	Strongly disagree
37/71 (52.1%)	31 /71 (43.7%)	2/71 (2.8%)	1/71 (1.4%)	0%
Agreement: 95.8%		2.8%	Disagreement: 1.4%	
***Rate of agreement: 94.4%***				
**2. It is comprehensive**				
Strongly agree	Agree	Indifferent	Disagree	Strongly disagree
29/71 (40.8%)	38/71 (53.6%)	2/71 (2.8%)	2/71 (2.8%)	0%
Agreement: 94.4%		2.8%	Disagreement: 2.8%	
***Rate of agreement: 91.6%***				
**3. It is correlated with patient's clinical presentation**				
Strongly agree	Agree	Indifferent	Disagree	Strongly disagree
26/71 (36.6%)	39/71 (55%)	4/71 (5.6%)	2/71 (2.8%)	0%
Agreement: 91.6%		5.6%	Disagreement: 2.8%	
***Rate of agreement: 88.8%***				
**4. It is correlated with patient's management**				
Strongly agree	Agree	Indifferent	Disagree	Strongly disagree
25/71 (35.2%)	38/71 (53.5%)	7/71 (9.9%)	1/71 (1.4%)	0%
Agreement: 88.7%		9.9%	Disagreement: 1.4%	
***Rate of agreement: 87.3%***				
**5. It is simple and users friendly**				
Strongly agree	Agree	Indifferent	Disagree	Strongly disagree
32/71 (45.1%)	27/71 (38.0%)	10/71 (14.1%)	2/71 (2.8%)	0%
Agreement: 83.1%		14.1%	Disagreement: 2.8%	
***Rate of agreement: 80.3%***				
**6. There is a smooth movement from the old proposals to the this new one**				
Strongly agree	Agree	Indifferent	Disagree	Strongly disagree
16/71 (22.5%)	38/71 (53.5%)	13/71 (18.4%)	4/71 (5.6%)	0%
Agreement: 76.0%		18.4%	Disagreement: 5.6%	
***Rate of agreement: 70.4%***				
**III. Which of the following statements are accomplished by the new system**				
**1. Anatomy is used correctly as the basic characteristic for patients’ grouping**				
Strongly agree	Agree	Indifferent	Disagree	Strongly disagree
35/71 (49.3%)	36/71 (50.7%)	0%	0%	0%
Agreement: 100.0%		0%	Disagreement: 0%	
***Rate of agreement: 100%***				
**2. Uterus as the ‘key’ organ of the female genital tract is used correctly in priority for the development of a new system**				
Strongly agree	Agree	Indifferent	Disagree	Strongly disagree
39/71 (55.0%)	29/71 (40.8%)	1/71 (1.4%)	1/71 (1.4%)	1/71 (1.4%)
Agreement: 95.8%		1.4%	Disagreement: 2.8%	
***Rate of agreement: 93%***				
**3. Embryological origin is not used, correctly, as the basic characteristic for patients’ grouping**				
Strongly agree	Agree	Indifferent	Disagree	Strongly disagree
18/71 (25.4%)	33/71 (46.5%)	17/71 (23.9%)	3/71 (4.2%)	0%
Agreement: 71.9%		23.9%	Disagreement: 4.2%	
***Rate of agreement: 67.7%***				
**4. Embryological origin is used successfully as a secondary characteristic for patients’ grouping**				
Strongly agree	Agree	Indifferent	Disagree	Strongly disagree
12/71 (16.9%)	42/71 (59.2%)	14/71 (19.7%)	2/71 (2.8%)	1/71 (1.4%)
Agreement: 76.1%		19.7%	Disagreement: 4.2%	
***Rate of agreement: 71.9%***				
**5. Uterine anomalies are classified successfully in the proposed V classes**				
Strongly agree	Agree	Indifferent	Disagree	Strongly disagree
23/71 (32.3%)	39/71 (55.0%)	7/71 (9.9%)	1/71 (1.4%)	1/71 (1.4%)
Agreement: 87.3%		9.9%	Disagreement: 2.8%	
***Rate of agreement: 84.5%***				
**6. The classification of fusion defects in one class (dysfused uterus/Class III) instead of two in the AFS classification (didelphys and bicornuate uterus) system is more functional and helps creating a more accurate and clear in definition category**				
Strongly agree	Agree	Indifferent	Disagree	Strongly disagree
21/71 (29.5%)	33/71 (46.5%)	9/71 (12.7%)	8/71 (11.3%)	0%
Agreement: 76.0%		12.7%	Disagreement: 11.3%	
***Rate of agreement: 64.7%***				
**7. The addition of normal uterus as Class 0 gives the opportunity to effectively classify cervical and/or vaginal only anomalies and, thus, obstructive anomalies**				
Strongly agree	Agree	Indifferent	Disagree	Strongly disagree
36/71 (50.7%)	26/71 (36.6%)	6/71 (8.5%)	3/71 (4.2%)	0%
Agreement: 87.3%		8.5%	Disagreement: 4.2%	
***Rate of agreement: 83.1%***				
**8. The independent classification of cervical and vaginal anomalies gives the opportunity to clearly classify the female's genital tract anomalies**				
Strongly agree	Agree	Indifferent	Disagree	Strongly disagree
32/71 (45.1%)	36/71 (50.7%)	2/71 (2.8%)	1/71 (1.4%)	0%
Agreement: 95.8%		2.8%	Disagreement: 1.4%	
***Rate of agreement: 94.4%***				
**9. Cervical and vaginal anomalies are classified successfully in the proposed four classes**				
Strongly agree	Agree	Indifferent	Disagree	Strongly disagree
20/71 (28.1%)	41/71 (57.8%)	8/71 (11.3%)	2/71 (2.8%)	0%
Agreement: 85.9%		11.3%	Disagreement: 2.8%	
***Rate of agreement: 83.1%***				
**IVa. Could you please report a case that, according to you, could not be effectively classified by this new system?**				
**IVb. Comments (feel free to make additional comments); please notice that it is important**				

The scale of answers includes five degrees to rank the agreement in each scientific issue; the extent of agreement between the participants is shown in percentages.

It should be noted that in the same questionnaire there were parts aiming to assess the opinion of the participants for the existing classification systems (their advantages and their limitations) and to have their comments on them; the results on this issue will be presented in another document.

The agreement rate for each statement was calculated as follows: *no. of participants who agree − no. of participants who disagree/no. of participants (agree* *+ indifferent*+ *disagree)*; agreement rates >67% are considered as consensus for agreement and <−67% as consensus for disagreement among the participants. Values between 50 and 67% are considered as indication of acceptance whereas between −50 and −67 as indication of rejection of the statement considered. Any value between 50 and −50% is considered as an expression of neutral opinion.

##### Selection of participants

The selection of participants in the DELPHI procedure was a crucial issue. The SC decided to remain within the European borders since it was a European project; an invitation to participate together with a selection questionnaire was sent to all members of the ESHRE SIG RS (Special Interest Group Reproductive Surgery), all the ESGE members and to selected well-known European experts in the field. Acceptance of the invitation is thought to be an expressed interest in the field of female genital anomalies and responders were included in the list of the participants of the DELPHI procedure.

In total, the invitation has been sent to 454 professionals; 118 of them responded, thus becoming the participants of the DEPLHI procedure.

#### DELPHI procedure: first round

DELPHI questionnaire has been sent to the 118 experts who accepted to participate in the procedure; participants were asked to rank their agreement with each statement in the questionnaire and to add their comments at the end.

In total, 89 (75.4%) participants responded to the questionnaire and made their comments. The main results of the first round were as follows (Table I):

- There was an agreement consensus among the participants that there was a need for a new classification system; agreement rate was as high as 82%. This confirms the initial feeling of the SC *that the target to develop a new classification system corresponds to the needs of the scientific community*.
- Concerning the relative importance of the characteristics that a new system has to fulfill, the ranking was as follows (selected as one of the three more important characteristics): first to be clear and accurate with 72.1%, second to be correlated with patient's management with 66.2% and third to be simple and user friendly with 64.7%. The smooth transition from the old systems (mainly that of AFS) to the new one was the last in the ranking preferences of the participants.
- Concerning the *selection of the basic characteristics* for patients' grouping in a new system, the results were as follows:
  - There was a high agreement consensus among the participants that ‘*anatomy should be used as the main characteristic*’ for the development of the new system with an agreement rate of 94.4%.
  - Participants did not support the use of *embryology* as the primary characteristic for patients' grouping with an agreement rate of 3.4%, but it seems that they could accept its *use as a secondary characteristic* with an agreement rate of 61.8%.
  - There was an indication of agreement among the participants that there is a *key organ* for patients' grouping with an agreement rate of 50.6% and most of them reported that this *is uterus*.

Thus, the main conclusions from the first round of the DELPHI procedure were that there was a need for a new classification system, which had to be clear and accurate in its definitions, correlated with patients' management and as simple as possible. The smooth transition from the old system, mainly that of AFS, to the new one was not important in the design and the acceptance of the new system. Furthermore, anatomy should be used as the basic characteristic for patients grouping. The degree of consensus among the participants regarding these statements was thought to be extremely high. Moreover, there was an indication that uterus could be used as the key organ in the design of system's categories and embryology could be used as a secondary characteristic for patients' grouping.

#### DELPHI procedure: second round

The results of the first round have been taken into account in the new proposal for the classification system prepared in the meantime. After its publication (Grimbizis and Campo on Behalf of the CONUTA Group, 2012), the SC decided to start the second round of the DELPHI procedure.

Before sending DELPHI questionnaire Round 2, the SC has sent the following to the participants: (i) the results of the first round and (ii) the SC's proposal for the classification of female genital malformations (Fig. 1); DELPHI questionnaire Round 2 (Tables II and III) has been sent 1 month later.

The participants have been asked to rank their agreement with each statement of the questionnaire and to add their comments at the end taking into account *the results of the first round and the new proposed classification system*.

In total, 71 (79.8%) participants responded. The main results of the second round were as follows (Tables II and III):

- There was an agreement consensus among the participants that the new system fulfills their needs and expectations with an agreement rate as high as 84.5%,
- More importantly, there was a high agreement consensus that the new proposed system seemed to be clear and accurate in its definitions with an agreement rate of 94.4%, comprehensive with an agreement rate of 91.6%, correlated with patients' clinical presentation with an agreement rate of 88.8% and correlated with patients' management with an agreement rate of 87.3%. Furthermore, there was also an agreement consensus that the new system seemed to be simple and user friendly with an agreement rate of 80.3%. Moreover, there was an agreement consensus that the new system gives the opportunity for a smooth transition from the old AFS system with an agreement rate of 70.4%, although this was not ranked as being important during the DELPHI first round.
- The third part of the structured questionnaire included questions aiming to evaluate the agreement of participants on the design of the new system and the grouping of patients; the new system incorporates a lot of radical changes in the way of categorization.
  - There was an absolute agreement consensus that ‘anatomy is used correctly as the basic characteristic in patients grouping’ with 100% agreement rate, an extremely high agreement consensus that ‘uterus is also correctly used as the key organ for the design of main classes’ with an agreement rate of 93% and a high agreement consensus that ‘uterine anomalies are classified successfully in the proposed V classes’ with an agreement rate of 84.5%; *the acceptance of these three points with such high rates is very important since they represent key concepts of the new system*.
  - There was an agreement consensus that ‘embryological origin is used successfully as a secondary characteristic for patients’ grouping’ with an agreement rate of 71.9%.
  - There was a high agreement consensus among the participants that ‘the addition of normal uterus as Class 0 gives the opportunity to effectively classify cervical and/or vaginal anomalies and, thus, obstructive anomalies’ with an agreement rate of 83.1%, an extremely high agreement consensus that ‘the independent classification of cervical and vaginal anomalies gives the opportunity to clearly classify female genital anomalies’ with an agreement rate of 94.4% and a high agreement consensus that ‘cervical and vaginal anomalies are successfully classified in the proposed four classes’ with an agreement rate of 83.1%; *acceptance of these concepts is also very important since they represent a radical change in the classification design of the new system*.
  - There was an indication of agreement (although not an agreement consensus) that ‘the classification of fusion defects in one class (dysfused uterus/Class III) instead of two (didelphys and bicornuate uterus) in the AFS classification system is more functional and helps creating a more accurate and clear definition category’ with an agreement rate of 64.7%. This point was another change in the design of the new system; taking into account the results of the DELPHI procedure and some comments of the responders, the members of the SC focused on the need to further clarify the terminology, the clinical concept and the definitions of this class and its sub-classes.
  - Furthermore, there were a lot of very useful comments from the participants, which were taken into account in the final version of the new system.

Thus, the main conclusion of the second round was that it was observed an agreement consensus among the participants for the new proposed system. However, there were some points that could be incorporated creatively in the classification of female genital anomalies.

### Consensus development

The final step in the creation of the new classification system was the development of consensus. For this reason, the results of the DELPHI procedure and the comments of the participants have been critically evaluated and the appropriate changes have been incorporated in the final version of the ESHRE/ESGE classification system. The main decisions of the SC in the process of consensus development were as follows:

- The results of the DELPHI procedure indicated that the proposal of the SC fulfilled the expectations of scientists and was accepted by them; hence, it was decided to keep the main concepts in the design and the classes of the new system.
- Together with the results of the DELPHI procedure for Class III (dysfused uterus), indicating the need for further clarification of its concepts and definitions, there was a comment pointing out that the term ‘dysfused’ is not commonly used. Examining this issue, the SC underlined that a descriptive term of the uterine gross morphology has been already adopted for most classes of the new system; on the contrary, the chosen term ‘dysfused’ is mainly an embryological one creating confusion. Thus, it was decided to change this term with one describing the morphology of the uterus and including in its meaning the former ‘bicornuate’ and ‘didelphys’ uterus; the term *bicorporeal* is adopted as it was deemed more appropriate. Furthermore, in order to keep the same principles in the classes terminology, the term ‘unilaterally formed uterus’ was changed to ‘hemi-uterus’.
- The ‘dysfused septate’ uterus resulting from a concomitant fusion and absorption defect was included as a clear subcategory (IIIc) of Class III as ‘bicorporeal septate uterus’.
- The absence of arcuate uterus from the new classification system has been commented by a lot of participants; some groups supported the notion that even very small deformities of the uterine cavity (arcuate uterus) could be associated with poor pregnancy outcome (Tomazevic *et al*., 2007; Gergolet *et al*., 2012). However, it was pointed out that until now the term arcuate was quite confusing including patients with different degrees of uterine deformity, even partial septa, since its definition is not clear at all. Hence, the necessity to have clear definitions was more than obvious based on the experience gained from the application of the AFS system. Thus, it was decided that septate uterus should remain a clear category including only patients with midline indentation of >50% of the uterine wall thickness. A new subcategory under the general term ‘others’ was added in Class I/dysmorphic uterus, giving the opportunity to include all minor deformities of endometrial cavity including midline indentations <50% of the uterine wall thickness; the clinical value of this variant needs further clinical research. Thus, definition of classes and especially those of septate uterus remained clear.
- There were some comments regarding the subcategories of cervical and vaginal anomalies. A proposal to include partial vaginal agenesis as a different subcategory has not been adopted since it does not seem to be of any clinical value. The term (cervical/vaginal) dysplasia was deleted from the classification since it is generally used for intraepithelial neoplasia. Thus, the subcategories remained as in the initial proposal.
- The simplicity of the new system has been discussed. It was pointed out that for the vast majority of anomalies only the use of uterine categories was necessary, while cervical and vaginal ones were not.

In conclusion, the ESHRE/ESGE classification system was the final result of a process including scientific research, consensus assessment and consensus development.

## The ESHRE/ESGE classification system

### Design of the new system: main concepts

**Figure 2:**
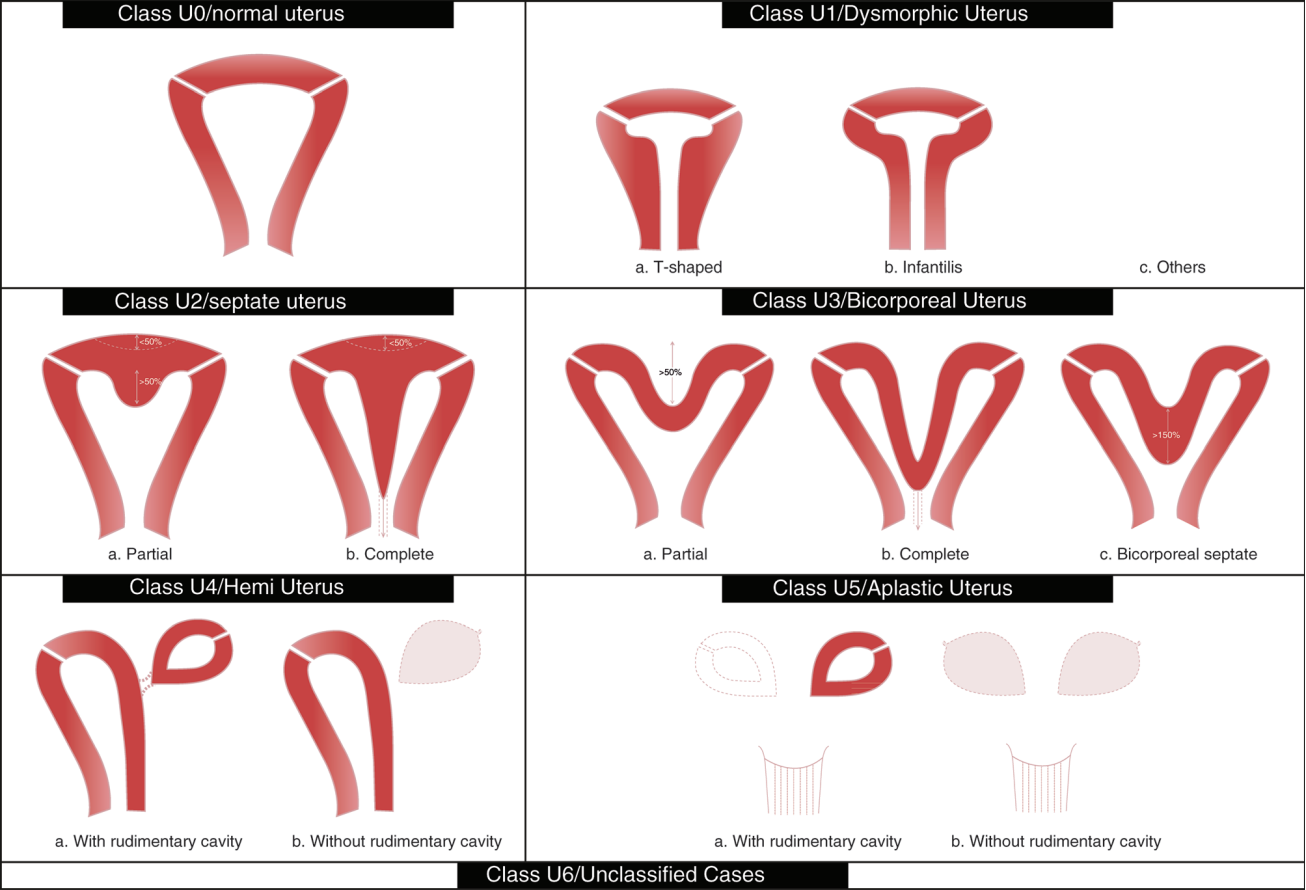
ESHRE/ESGE classification of uterine anomalies: schematic representation (Class U2: internal indentation >50% of the uterine wall thickness and external contour straight or with indentation <50%, Class U3: external indentation >50% of the uterine wall thickness, Class U3b: width of the fundal indentation at the midline >150% of the uterine wall thickness).

**Figure 3:**
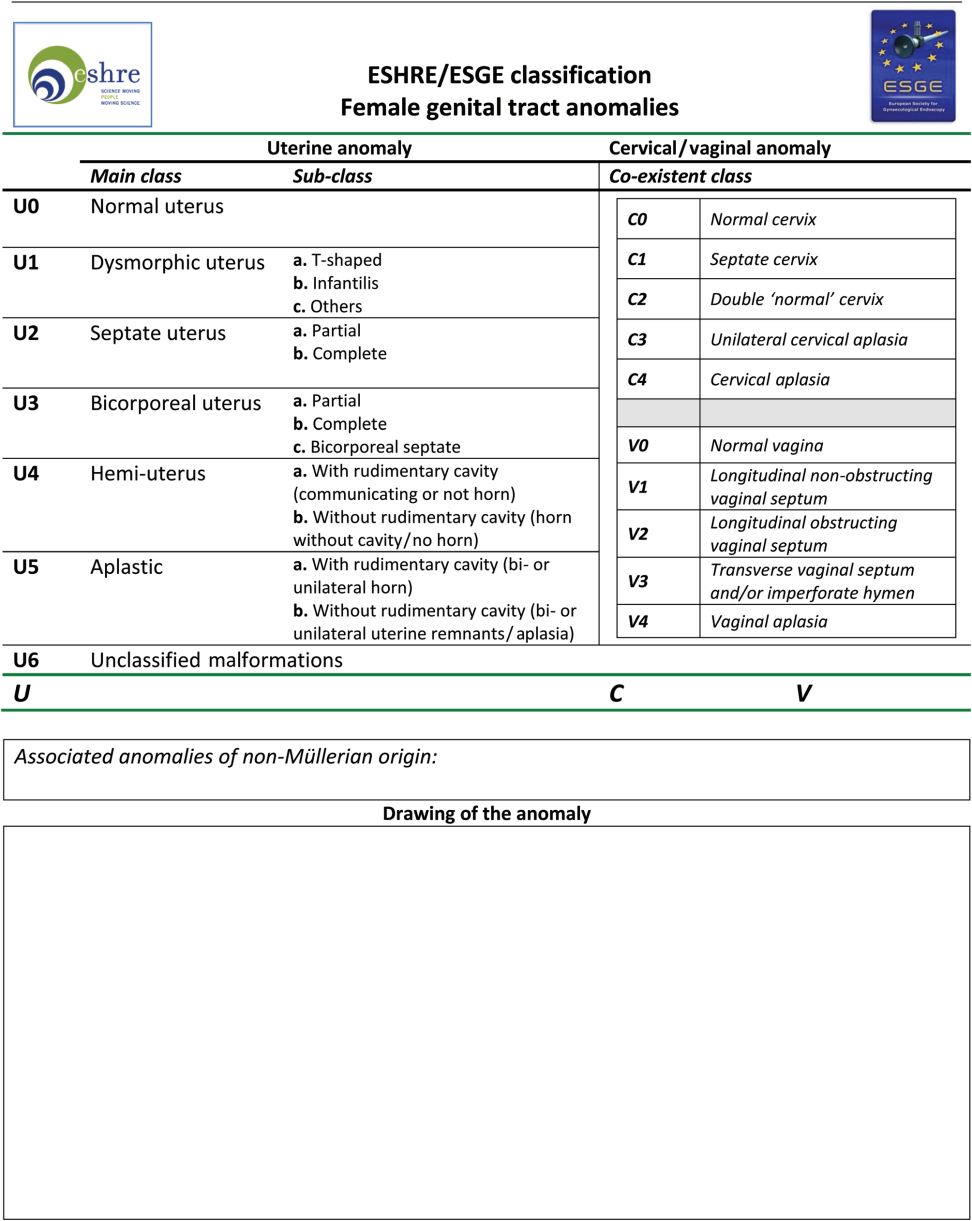
Scheme for the classification of female genital tract anomalies according to the new ESHRE/ESGE classification system.

The ESHRE/ESGE classification system is presented in Figs 2 and 3. It has the following general characteristics:

- Anatomy is the basis for the systematic categorization of anomalies.
- Deviations of uterine anatomy deriving from the same embryological origin are the basis for the design of the main classes.
- Anatomical variations of the main classes expressing different degrees of uterine deformity and being clinically significant are the basis for the design of the main sub-classes.
- Cervical and vaginal anomalies are classified in independent supplementary sub-classes.

Anomalies are sorted in the classes and sub-classes of the system according to increasing severity of the anatomical deviation; the less severe variants are placed in the beginning, the more deformed types at the end. For the sake of simplicity, an extremely detailed sub-classification is avoided: anatomical variations of uterine, cervical and vaginal anomalies are grouped in sub-classes having as criterion the clinical significance of the abnormality.

### Definitions: uterine main classes and sub-classes

- *Class U0* incorporates all cases with normal uterus. A normal uterus is any uterus having either straight or curved interostial line but with an internal indentation at the fundal midline not exceeding 50% of the uterine wall thickness. The use of absolute numbers (e.g. indentation of 5 mm) is avoided in definitions as uterine dimensions as well as uterine wall thickness could normally vary from one patient to another. Thus, it was decided to define uterine deformity as proportions of uterine anatomical landmarks (e.g. uterine wall thickness). The addition of normal uterus gives the opportunity to independently classify congenital malformations of the cervix and vagina (Rock *et al*., 2010; Grimbizis *et al*., 2004; Strawbrigde *et al*., 2007).
- *Class U1 or Dysmorphic uterus* incorporates all cases with normal uterine outline but with an abnormal shape of the uterine cavity excluding septa. Class I is further subdivided into three categories:
  - *Class U1a or T-shaped uterus* characterized by a narrow uterine cavity due to thickened lateral walls with a correlation 2/3 uterine corpus and 1/3 cervix.
  - *Class U1b or uterus infantilis* characterized also by a narrow uterine cavity without lateral wall thickening and an inverse correlation of 1/3 uterine body and 2/3 cervix.
  - *Class U1c or others* which is added to include all minor deformities of the uterine cavity including those with an inner indentation at the fundal midline level of <50% of the uterine wall thickness. This aims to facilitate groups who want to study patients with minor deformities and to clearly differentiate them from patients with septate uterus (Tomazevic *et al*., 2007; Gergolet *et al*., 2012). Usually, dysmorphic uteri are smaller in size.
- *Class U2 or septate uterus* incorporates all cases with normal fusion and abnormal absorption of the midline septum. Septate is defined as the uterus with normal outline and an internal indentation at the fundal midline exceeding 50% of the uterine wall thickness. This indentation is characterized as septum and it could divide partly or completely the uterine cavity including in some cases cervix and/or vagina (see cervical and vaginal anomalies).
  Class U2 is further divided into two sub-classes *according to the degree of the uterine corpus deformity*:
  - *Class U2a or partial septate uterus* characterized by the existence of a septum dividing partly the uterine cavity above the level of the internal cervical os
  - *Class U2b or complete septate uterus* characterized by the existence of a septum fully dividing the uterine cavity up to the level of the internal cervical os. Patients with complete septate uterus (Class U2b) could have or not cervical (e.g. bicervical septate uterus) and/or vaginal defects (see cervical/vaginal anomalies) (Grimbizis and Campo on Behalf of the CONUTA Group, 2012).
- *Class U3 or bicorporeal uterus* incorporates all cases of fusion defects. As bicorporeal is defined the uterus with an abnormal fundal outline; it is characterized by the presence of an external indentation at the fundal midline exceeding 50% of the uterine wall thickness. This indentation could divide partly or completely the uterine corpus including in some cases the cervix and/or vagina (see cervical and vaginal anomalies). As it could easily be imagined, it is also associated with an inner indentation at the midline level that divides the cavity as happens also in the case of septate uterus.
  Class U3 is further divided into three sub-classes *according to the degree of the uterine corpus deformity*:
  - *Class U3a or partial bicorporeal uterus* characterized by an external fundal indentation partly dividing the uterine corpus above the level of the cervix.
  - *Class U3b or complete bicorporeal uterus* characterized by an external fundal indentation completely dividing the uterine corpus up to the level of the cervix.
  - *Class U3c or bicorporeal septate uterus* characterized by the presence of an absorption defect in addition to the main fusion defect. In patients with bicorporeal septate uterus (Class U3c) the width of the midline fundal indentation exceeds by 150% the uterine wall thickness; these patients could be partially treated by hysteroscopic cross section of the septate element of the defect. It should be noted, also, that patients with complete bicorporeal uterus (Class U3b) could have or not co-existent cervical (e.g. double cervix/formerly didelphys uterus) and/or vaginal defects (e.g. obstructing or not vaginal septum).
- Class U4 or hemi-uterus incorporates all cases of unilateral formed uterus. Hemi-uterus is defined as the unilateral uterine development; the contralateral part could be either incompletely formed or absent. It is a formation defect; the necessity to classify it in a different class than that of aplastic uterus (formation defect) is due to the existence of a fully developed functional uterine hemi-cavity.
  Class U4 is further divided into two sub-classes *depending on the presence or not of a functional rudimentary cavity*;
  - *Class U4a or hemi-uterus with a rudimentary (functional) cavity* characterized by the presence of a communicating or non-communicating functional contralateral horn.
  - *Class U4b or hemi-uterus without rudimentary (functional) cavity* characterized either by the presence of non-functional contralateral uterine horn or by aplasia of the contralateral part. The presence of a functional cavity in the contralateral part is the only clinically important factor for complications, such as hemato-cavity or ectopic pregnancy in the rudimentary horn or hemato-cavity and treatment (laparoscopic removal) is always recommended even if the horn is communicating (Fedele *et al*., 2005; Theodoridis *et al*., 2006).
- Class U5 or aplastic uterus incorporates all cases of uterine aplasia (Aittomaki *et al*., 2001; Oppelt *et al*., 2012). It is a formation defect characterized by the absence of any fully or unilaterally developed uterine cavity. However, in some cases there could be bi- or unilateral rudimentary horns with cavity, while in others there could be uterine remnants without cavity (Oppelt *et al*., 2012). Treatment options in patients having rudimentary horn with cavity are not yet clear (Rall *et al*., 2013). Furthermore, it should be noted that patients with aplastic uterus could usually have co-existent defects (e.g. vaginal aplasia/Mayer-Rokitansky-Küster-Hauser syndrome) (Oppelt *et al*., 2012).
  Class U5 is further divided into two sub-classes *depending on the presence or not of a functional cavity in an existent rudimentary horn*:
  - *Class U5a or aplastic uterus with rudimentary (functional) cavity* characterized by the presence of bi- or unilateral functional horn,
  - *Class U5b or aplastic uterus without rudimentary (functional) cavity* characterized either by the presence of uterine remnants or by full uterine aplasia. The presence of a horn with cavity is clinically important and it is used as a criterion for sub-classification because it is combined with health problems (cyclic pain and/or hemato-cavity) necessitating treatment.
- *Class U6* is kept for still unclassified cases. Modern imaging technology (ultrasound and/or magnetic resonance imaging) could provide objective estimations of uterine anatomy for the needs of differential diagnosis among the six groups. However, infrequent anomalies, subtle changes or combined pathologies could not be allocated correctly to one of the six groups. A sixth class was created for these cases in order to keep the other groups ‘clear’. Furthermore, the system is designed to include, hopefully, all cases resulting from formation, fusion or absorption defects of normal embryological development. Duplication defects or ectopic Müllerian tissue anomalies, if existing, could not be described; these anomalies could be put in this class.

### Definitions: co-existent cervical anomalies

- *Sub-class C0 or normal cervix* incorporates all cases of normal cervical development.
- *Sub-class C1 or septate cervix* incorporates all cases of cervical absorption defects. *It is characterized by the presence of a normal externally rounded cervix with the presence of a septum*.
- *Sub-class C2 or double cervix* incorporates all cases of cervical fusion defects. *It is characterized by the presence of two distinct externally rounded cervices; these two cervices could be either fully divided or partially fused*. It could be combined with a complete bicorporeal uterus as a Class U3b/C2 in the formerly didelphys uterus.
- *Sub-class C3 or unilateral cervical aplasia* incorporates all cases of unilateral cervical formation. *It is characterized by the unilateral, only, cervical development; the contralateral part could be either incompletely formed or absent.* Obviously, this has happened in Class U4 patients; however, this is not necessary to be mentioned in the final classification report (Class U4 instead of Class U4/C3) as being apparent. On the other hand, this sub-class gives the opportunity to classify other seldom anomalies such as complete bicorporeal uterus with unilateral cervical aplasia as Class U3b/C3, which is a severe obstructing anomaly.
- *Sub-class C4 or cervical aplasia* incorporates all cases of complete cervical aplasia but, also, those of severe cervical formation defects. *It is characterized either by the absolute absence of any cervical tissue or by the presence of severely defected cervical tissue such as cervical cord, cervical obstruction and cervical fragmentation.* The decision to include all variants of cervical dysgenesis in sub-class C4 was made in order to avoid an extremely extensive sub-classification, which does not seem to be user friendly. This sub-class could be combined with a normal or a defected uterine body and gives the opportunity to classify all obstructing anomalies due to cervical defects.

### Definitions: co-existent vaginal anomalies

- *Sub-class V0 or normal vagina* incorporates all cases of normal vaginal development.
- *Sub-class V1 or longitudinal non-obstructing vaginal septum*. The incorporated anomaly in this sub-class is clear; it gives the opportunity to classify variants of septate or bicorporeal uteri together with septate or double cervices.
- *Sub-class V2 or longitudinal obstructing vaginal septum*. The incorporated anomaly in this sub-class is also clear and, its utility for the effective classification of obstructing anomalies due to vaginal defects is obvious.
- *Sub-class V3 or transverse vaginal septum and/or imperforate hymen*. This sub-class incorporates obviously different vaginal anomalies and their variants (mainly those of transverse vaginal septa); this was decided in order to avoid an extremely extensive sub-classification for the classification system's simplicity. The decision to put together those vaginal anomalies in this sub-class is due to the fact that they are usually present as isolated vaginal defects and they have the same clinical presentation (obstructing anomalies).
- Sub-class V4 or vaginal aplasia incorporates all cases *of complete or partial vaginal aplasia*.

## Concluding remarks

The CONUTA ESHRE/ESGE Working Group for the study of congenital malformations of the female genital tract presents the new ESHRE/ESGE classification system. The development of the system was based on the scientific work of critical review of the advantages and disadvantages of current proposals, on the use of the DELPHI procedure for consensus assessment among the scientists working in this field and on consensus development by the SC of this project. The use of the DELPHI procedure gave the opportunity to a large number of scientists to participate. Almost 90 participants took part in the process of development of the new classification system, contributing with their structured answers and comments. A new clinical approach for the classification of uterine anomalies is proposed.

Uterine anatomy is the basis of the new system. Embryological origin has been adopted as the secondary basic characteristic in the design of the main classes. Cervical and vaginal anomalies are classified in independent co-existent sub-classes. It seems that the new system fulfils the needs and expectations of a large group of experts in the field. *Clinicians could use Fig. 3 for an easy and precise description of anomalies and they could also draw the scheme of the malformation*. The ESHRE/ESGE classification system of female genital anomalies could also be used as a starting point for the development of guidelines for their diagnosis and treatment.

## Authors’ roles

Scientific coordinators: G.G. and R.C.; Delphi procedure: S.G.(running) and H.B. (quality control); ESHRE: L.G. (Executive Committee) and V.T. (Special Interest Group of Reproductive Surgery); ESGE: S.B. (Executive Committee) and S.G. (Special Interest Group of Reproductive Surgery); Scientific Committee: S.B., R.C., C.D., A.D., S.G., M.G., G.G., T.-C.L. and V.T.; All the authors contributed to the review of the manuscript.

## Funding

This project was supported financially by both ESHRE and ESGE.

## Conflict of interest

None declared.
